# Development and validation of the nomogram of high fascial compartment pressure with pilon fracture

**DOI:** 10.1007/s00264-024-06402-2

**Published:** 2025-01-08

**Authors:** Xin Hu, Peiyuan Wang, Chengsi Li, Lin Liu, Xin Wang, Lin Jin, Kuo Zhao, Ling Wang, Zhiyong Hou

**Affiliations:** 1 Department of Orthopaedic Surgery, The Third Hospital of Hebei Medical University, Shijiazhuang, Hebei People’s Republic of China; 2 Orthopaedic Research Institute of Hebei Province, Shijiazhuang, Hebei 050051 People’s Republic of China; 3 NHC Key Laboratory of Intelligent Orthopaedic Equipment (The Third Hospital of Hebei Medical University), Shijiazhuang, People’s Republic of China; 4 Department of Orthopedic Oncology, The Third Hospital of Hebei Medical University, Shijiazhuang, Hebei People’s Republic of China

**Keywords:** High fascial compartment pressure, Acute compartmental syndrome, Pilon fracture, Predictors, Nomogram, Systemic immune-inflammation index

## Abstract

**Purpose:**

High Fascial Compartment Pressure (HCP) is one of the most common complications in ankle fractures. This study aimed to investigate the incidence of HCP in pilon fracture and analyze the risk factors of HCP in order to closely monitor its further development into Acute Compartment Syndrome. A nomogram is constructed and validated to predict HCP in patients with pilon fracture.

**Methods:**

We collected information on 1,863 patients with pilon fracture in the 3rd Hospital of Hebei Medical University Hospital from January 2019 to March 2024. Patients with HCP were assigned to the HCP group and those without HCP to the non-HCP group. The inpatient medical record system was inquired for data collection, including demographics, comorbidities, injury types, and laboratory biomarkers. Variables with a significance level of *P* < 0.05 in the univariate analysis were included in the multivariate logistic regression analysis. The backward stepwise regression method was applied to identify independent risk factors associated with HCP. The selected predictors were then entered into R software for further analysis, and Nomogram construction.

**Results:**

The rate of HCP was 11.57%. Several predictors of HCP were found, including Body Mass Index (BMI) (*p*<0.001), Deep Vein Thrombosis (*p* < 0.001), occurrence of Fracture Blister (FB) (*p* < 0.001), use of Dehydrating Agent (*p* < 0.001), duration of limb immobilization (*p* < 0.001),and Systemic Immune-inflammation Index (SII) (*p* < 0.001). In addition, BMI (*p* < 0.001, OR 1.52, 95% CI 1.35 to 1.71), DVT (*p* < 0.001, OR 4.35, 95% CI 2.51 to 7.52), duration of limb immobilization (*p* < 0.01, OR 1.66, 95%CI 1.25 to 2.20) and SII (*p* < 0.01, OR 1.00, 95%CI 1.00 to 1.00) were correlated with increased HCP risk. Meanwhile, FB (*p* < 0.001, OR 0.23, 95% CI 0.13 to 0.39) and Dehydrating Agent (*p* < 0.001, OR 0.10, 95% CI 0.06 to 0.19) were associated with decreased HCP risk. The nomogram was established based on six predictors independently related to HCP.

**Conclusions:**

Our investigation has shown that, compared with tibial diaphyseal fractures, pilon fractures are more prone to HCP because of their high energy injury characteristics. This research also shows BMI, DVT, occurrence of FB, use of Dehydrating Agent, duration of limb immobilization, and SII are independent risk factors for HCP in patients with pilon fracture. We have also devised a nomogram grounded in these identified predictors. In particular, this study found for the first time that SII is an independent risk factor for HCP, which provides a basis for clinical and basic science research on fascial immunology in the future.

## Introduction

The term “pilon fracture” lacks a precise definition, however, it typically refers to the fracture occurring in the distal third of the tibia, encompassing the articular surface of the distal tibia [1]. Tibial pilon fractures are quite common, accounting for 3%~10% of all tibial fractures [2–4]. Men exhibit a slightly higher incidence of these injuries compared to women, with the majority of cases occurring around the age of 45 [4, 5]. In 75%~90% of all cases the fibula is also fractured [6]. The generally unfavourable functional outcome, particularly high fascial compartment pressure (HCP), following high-energy tibial pilon fractures underscores the severity of the injury. In 2003, Pollak et al. conducted an extensive retrospective cohort analysis of pilon fractures utilizing the SF-36 score, a questionnaire assessing health-related quality of life. They discovered that scores were markedly lower in the injured cohort compared to an age-matched uninjured population, and even lower than those observed in patients with chronic conditions such as AIDS, diabetes or asthma [7].

HCP can precipitate Acute Compartment Syndrome (ACS), an orthopaedic emergency posing a threat to limb viability. ACS is typified by heightened fascial compartment pressure, leading to compromised microcirculation [8]. Tissue ischemia, resulting from compromised blood flow, can progress and contribute to the onset of rhabdomyolysis and irreversible myonecrosis within hours following the onset of symptoms [9, 10]. Recently, several scholars have proposed a new predictive index, referred to as the systemic immune-inflammation index (SII). SII uses neutrophils, lymphocytes and platelets, which can better reflect the inflammation and immune status of a patient’s body [11]. Nevertheless, there have been no reports on the correlation between SII and HCP following pilon fractures. The aim of the present study was to investigate the cross-sectional correlation between SII and HCP after pilon fractures, with the objective of identifying effective and readily available indicators that can promptly identify high-risk patients and aid in clinical decision-making.

Although extensive research and retrospective database analyses have been conducted on lower limb injuries [12–16], particularly in the context of ankle fractures, the clarity of such studies diminishes when focusing on pilon fractures. In particular, as a common type of ankle fracture, there has been a scarcity of research on the relationship between pilon fracture and HCP. As such, the aim of the present study was to provide a better understanding of the incidence, risk factors, and development of a prediction model for HCP in pilon fractures.

## Materials and methods

### Ethics statement

To effectively execute the present study, a systematic investigation was conducted into the electronic medical records of all patients diagnosed and treated for lower extremity fractures at our Hospital from January 2019 to March 2024.

### General information

**Fig. 1:**
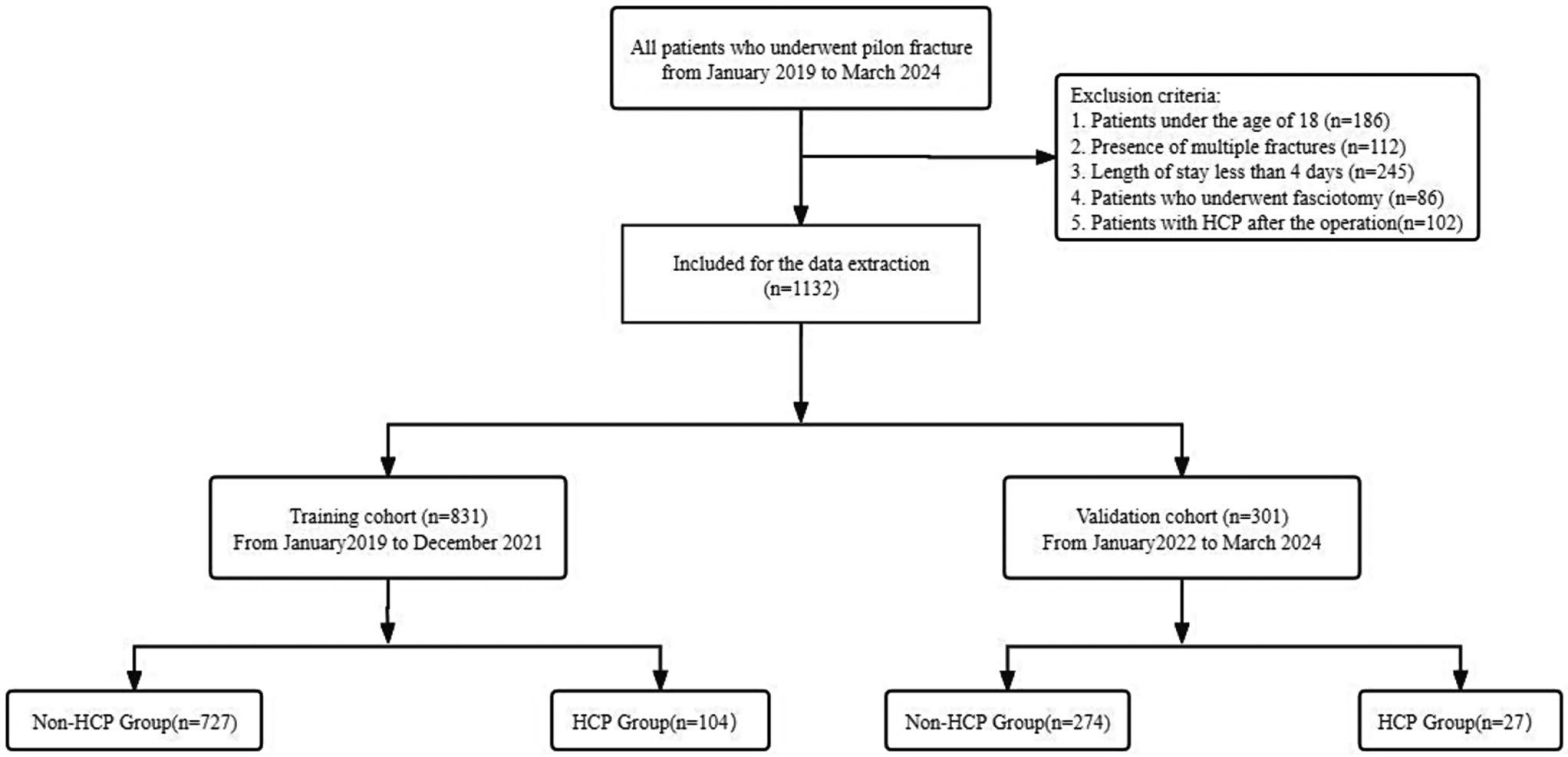
Patients selection flowchart

The aim of the present retrospective study was to compile clinical data from patients diagnosed with pilon fractures who were admitted to our hospital between January 2019 and March 2024. The inclusion criteria were as follows: (1) Patients aged 18 years or older; (2) Confirmation of pilon fracture through X-ray examination; (3) Tissue perfusion pressure < 30mmHg or patients with 5Ps. The exclusion criteria were as follows: (1) Patients under the age of 18; (2) Presence of multiple fractures; (3) Length of hospital stay less than 4 days; (4) Patients who underwent fasciotomy; (5) Patients with HCP after the operation (Fig. 1).

## Methods

Data on patient demographics, comorbidities, and preoperative laboratory examinations were systematically collected. The demographic variables encompassed age, gender, body mass index (BMI), fracture types, presence of deep vein thrombosis (DVT), occurrence of fracture blister (FB), Elixhauser comorbidity index (ECI), systemic immune-inflammation index (SII), smoking history, alcohol consumption, injury mechanism, time from injury to admission, use of a dehydrating agent, and duration of limb immobilization.

Additionally, a thorough investigation was conducted to assess various preoperative laboratory indicators, including albumin (ALB), alkaline phosphatase (ALP), aspartate aminotransferase (AST), alanine transaminase (ALT), calcium (Ca), potassium (K), sodium (Na), magnesium (Mg), phosphorus (P), chloride (Cl), globulin (GLOB), cholinesterase (CHE), creatine kinase (CK), creatinine (CREA), direct bilirubin (DBIL), glucose (GLU), lactic dehydrogenase (LDH), triglycerides (TG), total cholesterol (TC), total carbon dioxide (TCO2), ureophil (UREA), uric acid (UA), basophil (BAS), eosinophil (EOS), hematocrit (HCT), hemoglobin (HGB), lymphocyte (LYM), mean corpuscular hemoglobin concentration (MCHC), monocyte (MON), mean platelet volume (MPV), neutrophil (NEU), creatine kinase isoenzyme (CKMB), platelet (PLT), red blood cells (RBC), and white blood cells (WBC).

### Routine management of patients with pilon fracture

For open fractures, antibiotics were used to prevent infection (cefazolin sodium was routinely used, with clindamycin employed for patients with cephalosporin allergies). In addition, oral rivaroxaban or subcutaneous injection of low molecular weight heparin was performed to prevent DVT (in cases of DVT, the anticoagulant dose was escalated from prophylactic to therapeutic levels). For all patients suspected of having HCP, diagnosis was conducted using the STIC Monitor (Stryker Orthopaedics, Mahwah, NJ) or assessment for the presence of 5Ps symptoms(pain, paresthesia, paralysis, pulselessness and pallor). Blood indexes in the present study were acquired from the initial blood collection performed following the diagnosis of HCP.

### Statistical analysis

In the present study, SPSS software (version 25.0, SPSS Inc., New York, USA) was utilized, and *p* < 0.05 was considered statistically significant. The normality of continuous variables was assessed using the Shapiro-Wilk test. If normality was met, these variables were usually presented as mean ± SD (standard deviation) and analyzed using the t-test. However, if the assumption of normality was not met, the Mann-Whitney U test was employed instead. For categorical variables, which were represented by numerical counts and percentages, statistical analysis was conducted using the Chi-square test and Fisher’s exact test to evaluate differences between groups.

Variables with a significance level of *P* < 0.05 in the univariate analysis were included in the multivariate logistic regression analysis. The backward stepwise regression method was employed to identify independent risk factors associated with FB. The selected predictors were then input into R software (Version 3.6.5, R Foundation for Statistical Computing, Vienna, Austria) for further analysis. The “rms” package was employed to develop the nomogram.

The predictive efficacy and practical utility of the model were assessed using various evaluation metrics, including the Conformance index (C-index), receiver operating characteristic (ROC) curve, calibration curve, and decision curve analysis (DCA). The C-index, spanning from 0.5 to 1.0, and the area under the receiver operating characteristic curve (AUC) were indicative of the model’s discriminative capacity and predictive accuracy. These metrics were illustrated through the nomogram.

Calibration curves were employed to assess the concordance between diagnosed FB and predicted FB. A greater alignment between the predicted curve and the ideal curve indicated a stronger correspondence between the predicted probability and the actual probability. The clinical utility of the nomogram model was assessed using Decision Curve Analysis (DCA), which integrates threshold probabilities and net benefit to evaluate its practical application. To further validate the predictive performance of the model, the Bootstrap method was utilized for internal validation, and the modified C-index was calculated based on 1000 iterations of sampling. Subsequently, the validation set was utilized for additional external validation, and the performances of ROC curves, calibration curves, and DCA of the nomogram in both the training and validation cohorts were compared. A significance level of 0.05 was applied to all analyses.

## Results

A total of 1,863 cases were reviewed, with 731 cases being excluded from the study. In total, 1,132 cases met the study criteria with 1002 males and 130 females. The incidence of HCP in patients with pilon fractures was observed in 11.57% of cases, affecting 131 patients, while 1001 remained unaffected. The validation cohort comprised 301 cases.whereas the training cohort consisted of 831 cases, as detailed in Table 1. The statistical analysis indicated significant differences between the HCP and non-HCP groups with respect to several variables, including BMI (*p* < 0.001), the presence of DVT (*p* < 0.001), the presence of FB (*p* < 0.001), closed fractures (*p* < 0.001), time from injury to admission (*p* < 0.001), smoking history (*p* < 0.001), the administration of dehydrating agents (*p* < 0.001), ECI (*p* < 0.001), duration of limb immobilization (*p* < 0.001),and SII (*p* < 0.001). Specifically, individuals in the HCP group demonstrated higher BMI levels compared to those in the non-HCP group. Moreover, compared to the non-HCP group, patients in the HCP group exhibited a higher susceptibility to developing DVT or sustaining closed fractures. Additionally, the administration of dehydrating agents was associated with a reduced likelihood of HCP development. Moreover, individuals in the HCP group were more likely to have a history of smoking. Conversely, patients without a history of FB were more prone to developing HCP, and the HCP group experienced a longer duration from injury to admission. Finally, the HCP group exhibited a higher ECI and SII. In contrast, there were no statistically significant differences observed between the two groups in other variables (all *p* > 0.05).

**Table 1:** General information in training cohort patients

Variable	Non-HCP group (*n* = 727)	HCP group (*n* = 104)	*P*
Gender, n (%)			0.635
Female	78 (10.7%)	9 (8.7%)	
Male	649 (89.3%)	95 (91.3%)	
Age (years)	44.0 ± 16.8	43.8 ± 17.4	0.924
BMI (kg/m2)	24.3 ± 2.6	25.2 ± 2.3	< 0.001
DVT, n (%)			< 0.001
No	640 (88%)	65 (62.5%)	
Yes	87 (12%)	39 (37.5%)	
Fracture blister, n (%)			< 0.001
No	187 (25.7%)	51 (49%)	
Yes	540 (74.3%)	53 (51%)	
Open fracture, n (%)			< 0.001
No	286 (39.3%)	68 (65.4%)	
Yes	441 (60.7%)	36 (34.6%)	
Mechanism of injury, n (%)			0.248
Flat land tumble	228 (31.4%)	28 (26.9%)	
Traffic injury	252 (34.7%)	32 (30.8%)	
High falling	247 (34%)	44 (42.3%)	
Time from injury to admission, n (%)			< 0.001
< 1	450 (61.9%)	18 (17.3%)	
1–6	183 (25.2%)	16 (15.4%)	< 0.001
≥ 7	94 (12.9%)	70 (67.3%)	
Alcohol history, n (%)			0.894
No	222 (30.5%)	33 (31.7%)	
Yes	505 (69.5%)	71 (68.3%)	
Smoking history, n (%)			< 0.001
No	486 (66.9%)	47 (45.2%)	
Yes	241 (33.1%)	57 (54.8%)	
Dehydrating agent, n (%)			< 0.001
No	275 (37.8%)	68 (65.4%)	
Yes	452 (62.2%)	36 (34.6%)	
duration of limb immobilization	2.6 ± 1.0	3.1 ± 0.8	< 0.001
ECI	3.4 ± 1.1	7.0 ± 1.9	< 0.001
SII	991.8 ± 434.1	1271.4 ± 571.8	< 0.001

A comparison of laboratory test results between the two groups is outlined in Table 2. Compared to the non-HCP group, the HCP group exhibited significantly higher levels of MPV (*p* < 0.001), NEU (*p* < 0.001), WBC (*p* < 0.007), ALP (*p* < 0.001), CHE(*p* = 0.001), CREA (*p* < 0.001), GLOB (*p* < 0.001), K (*p* = 0.046), P (*p* = 0.002), TG (*p* < 0.001), and TP (*p* < 0.001). Conversely, HCT (*p* < 0.001), LYM (*p* = 0.016), CKMB (*p* < 0.001), PLT (*p* < 0.001), ALT (*p* = 0.016), Na (*p* = 0.001), ALB (*p* < 0.001), CL (*p* < 0.001), TC (*p* < 0.001), and TCO_2_ (*p* < 0.001) in the HCP group were lower compared to the non-HCP group. Nevertheless, no significant differences were observed in other laboratory data between these two groups (all *p* > 0.05).

**Table 2:** Laboratory results of patients

Variable	Non-HCP group(*n* = 727)	HCP group(*n* = 104)	*P*
BAS	0.1 ± 0.1	0.1 ± 0.1	0.128
EOS	37.1 ± 3.2	36.1 ± 2.5	< 0.001
HCT	122.1 ± 14.8	122.8 ± 9.7	0.531
HGB	1.5 ± 0.3	1.4 ± 0.3	0.016
LYM	0.9 ± 0.2	0.9 ± 0.2	0.805
MON	8.7 ± 0.6	8.9 ± 0.7	< 0.001
MPV	6.9 ± 3.2	9.7 ± 2.6	< 0.001
NEU	97.0 ± 55.3	68.5 ± 31.0	< 0.001
CKMB	219.9 ± 41.8	176.4 ± 26.2	< 0.001
PLT	5.4 ± 1.9	5.3 ± 1.8	0.697
RBC	14.6 ± 2.1	15.7 ± 4.0	0.007
WBC	35.8 ± 2.8	34.5 ± 1.4	< 0.001
ALB	66.5 ± 8.5	68.4 ± 3.7	< 0.001
ALP	46.3 ± 14.6	43.4 ± 11.1	0.016
ALT	80.7 ± 47.0	76.6 ± 34.7	0.287
AST	2.1 ± 0.1	2.1 ± 0.1	0.831
Ca	6.2 ± 0.6	6.4 ± 0.7	0.001
CHE	2591.3 ± 2077.8	2547.4 ± 1570.9	0.799
CK	104.5 ± 1.7	102.6 ± 2.6	< 0.001
CL	68.6 ± 5.9	71.3 ± 3.6	< 0.001
CREA	4.8 ± 1.2	5.0 ± 0.7	0.118
DBIL	21.1 ± 1.5	23.2 ± 1.4	< 0.001
GLOB	7.6 ± 1.1	7.7 ± 0.9	0.422
GLU	4.0 ± 0.4	4.1 ± 0.5	0.046
K	605.8 ± 317.8	629.2 ± 164.5	0.244
LDH	0.8 ± 0.1	0.8 ± 0.1	0.065
Mg	138.2 ± 2.8	137.2 ± 2.4	0.001
Na	1.1 ± 0.2	1.1 ± 0.1	0.002
P	3.2 ± 0.5	3.1 ± 0.3	< 0.001
TC	23.6 ± 1.8	23.1 ± 0.8	< 0.001
TCO_2_	1.2 ± 0.3	1.2 ± 0.2	< 0.001
TG	56.8 ± 4.8	58.5 ± 4.3	< 0.001
TP	314.0 ± 46.9	308.8 ± 21.5	0.057
UA	5.3 ± 0.8	5.2 ± 0.6	0.127
UREA	0.1 ± 0.1	0.1 ± 0.1	0.128

The stepwise regression logistic analysis identified significant factors related to HCP occurrence. BMI (*p* < 0.001, OR 1.52, 95% CI 1.35 to 1.71), DVT (*p* < 0.001, OR 4.35, 95% CI 2.51 to 7.52), duration of limb immobilization (*p* < 0.01, OR 1.66, 95%CI 1.25 to 2.20), and SII (*p* < 0.01, OR 1.00, 95%CI 1.00 to 1.00) were correlated with increased HCP risk. Meanwhile, FB (*p* < 0.001, OR 0.23, 95% CI 0.13 to 0.39) and dehydrating agent (*p* < 0.001, OR 0.10, 95% CI 0.06 to 0.19) were associated with decreased HCP risk. Further, as presented in Table 3, no additional factors influencing the incidence of HCP in these patients were identified.

**Table 3:** Binary logistic regression analysis of variables associated with HCP

Characteristics	OR	95% CI	*P*
BMI	1.52	1.35 ~ 1.71	< 0.001
DVT	4.35	2.51 ~ 7.52	< 0.001
Fracture blister	0.23	0.13 ~ 0.39	< 0.001
Dehydrating agent	0.10	0.06 ~ 0.19	< 0.001
duration of limb immobilization	1.66	1.25–2.20	< 0.001
SII	1.00	1.00 ~ 1.00	< 0.001

**Fig. 2:**
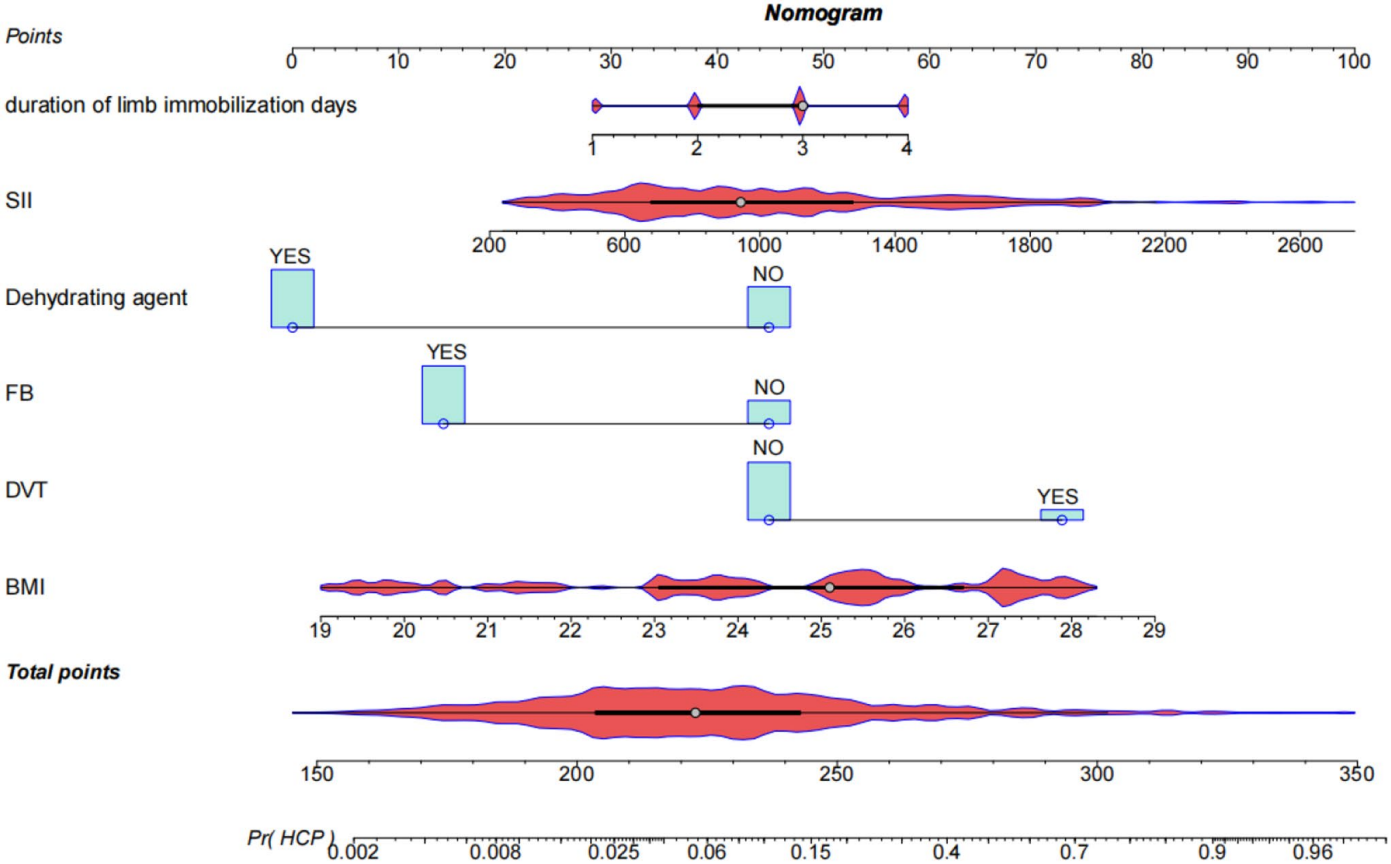
The nomogram designed for predicting the risk of HCP

**Fig. 3:**
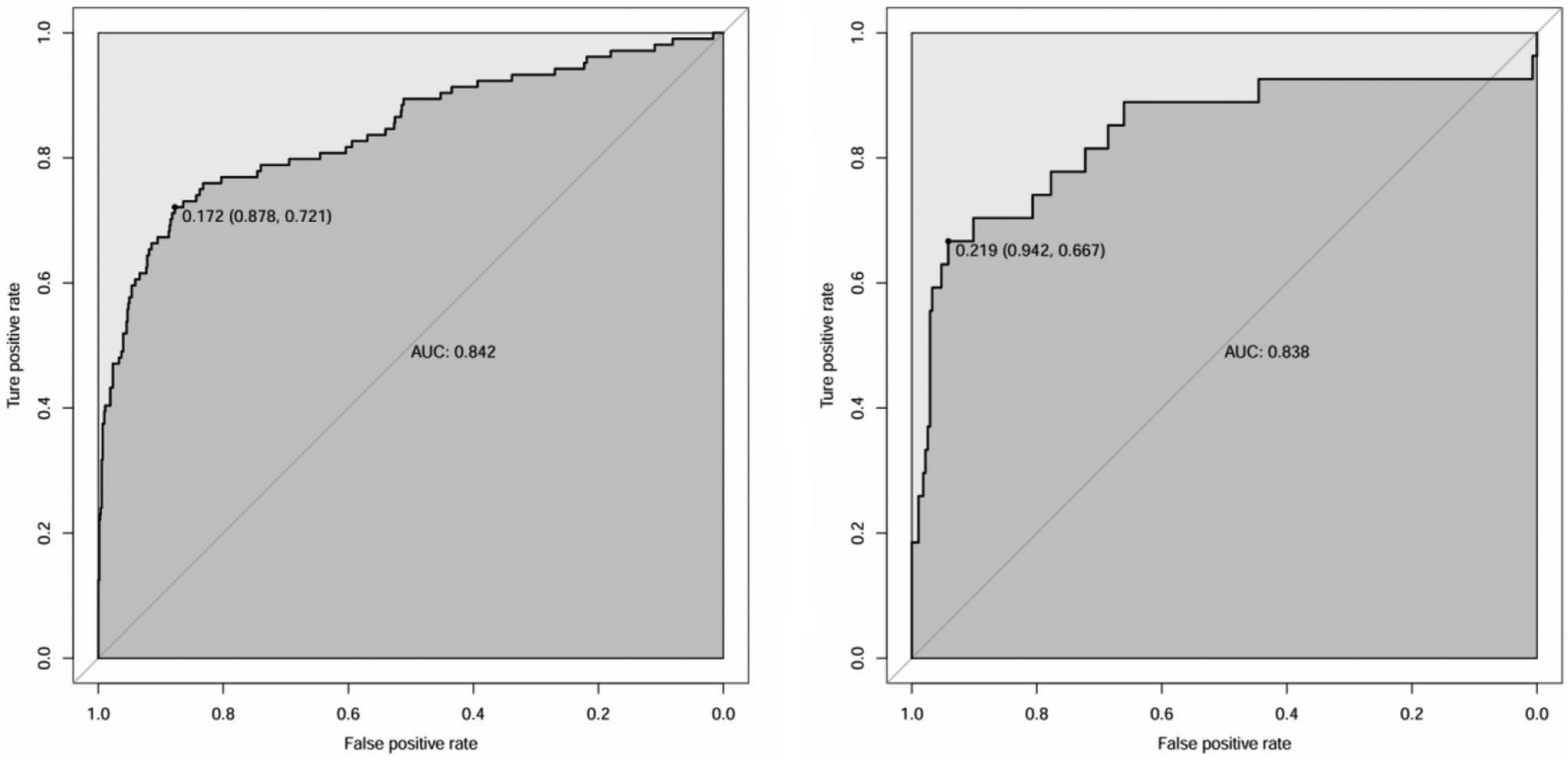
Receiver-operating characteristic (ROC) curves for the nomogram in the training (**a**) and validation sets (**b**)

The results were translated into a nomogram aimed at predicting the risk of HCP (see Fig. 2). Subsequently, a thorough assessment of the nomogram’s performance and reliability was undertaken. The predictive accuracy of the nomogram was positively correlated with the area under the curve (AUC). The AUC of the nomogram was 0.842 and 0.838 in the training and validation sets, respectively, The AUC for the predictive model exhibited strong discriminatory capability, measuring 0.842 (95% CI: 0.7931–0.8907) (Fig. 3), with a sensitivity of 72.1% and specificity of 87.8%.

**Fig. 4:**
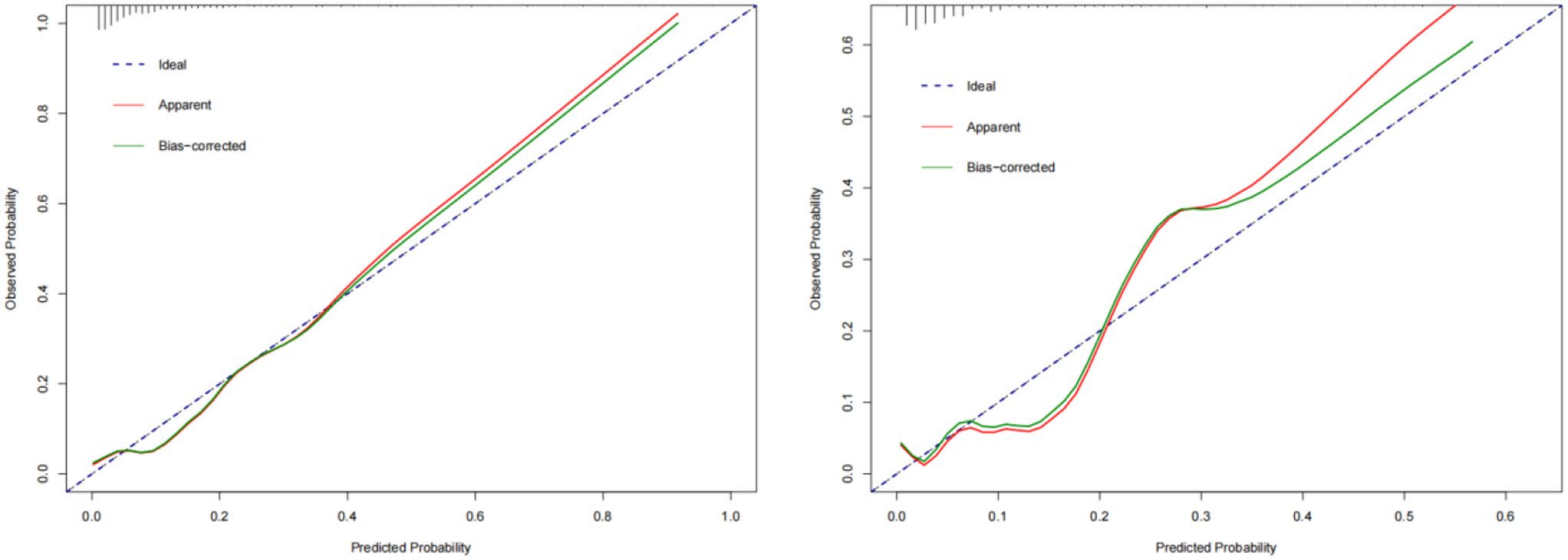
Calibration curves of nomogram in the training set (**a**) and validation set (**b**)

**Fig. 5:**
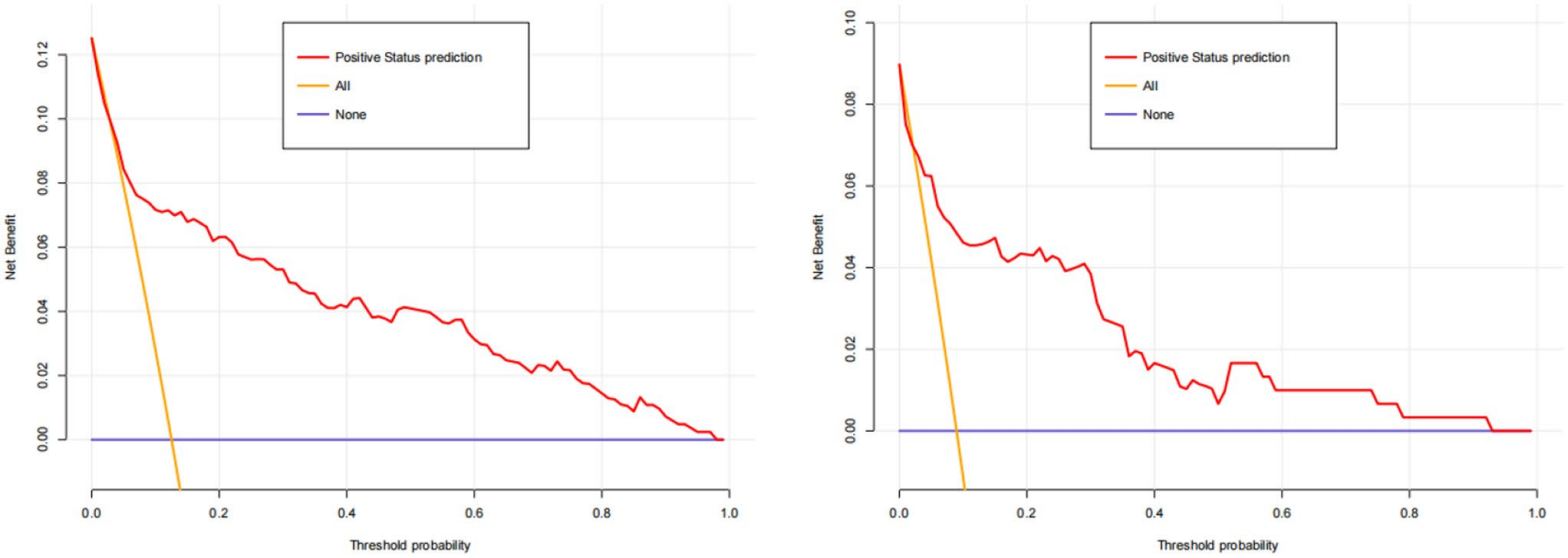
Decision curve analysis (DCA) of nomogram in the training set (**a**) and validation set (**b**)

These metrics highlight the effectiveness of the model in predicting risk. The nomogram achieved a C-index of 0.834, which remained consistent with an adjusted value of 0.806 after 1,000 bootstrap validations, indicating robust model performance and reliability. Additionally, the calibration curves (Fig. 4) demonstrated good agreement between the predicted and observed probabilities of HCP in trauma patients according to the nomogram. Moreover, the DCA for the nomogram revealed a positive net benefit compared to no intervention, particularly within the threshold probability range of 2–97% (Fig. 5). The described findings underscore the practical utility of the HCP prediction nomogram in guiding clinical decision-making.

As depicted in Fig. 2, a nomogram encompassing six covariates was constructed. To utilize the nomogram effectively: firstly, the subject’s BMI (expressed in kg/m^2^) was located on the corresponding variable axis. Then, a vertical line was drawn to intersect with the “Points” axis to obtain the corresponding score. This process was iterated for each covariate, and the scores were cumulatively totaled. The cumulative score was then matched with the corresponding point on the “Total Points” axis, and a vertical line was drawn to intersect with the “Risk of HCP” axis to determine the probability of developing HCP.

## Discussion

To the present knowledge, the present study represents the first attempt to develop and validate a nomogram specifically tailored to predict the risk of HCP in patients with isolated pilon fractures. The present investigation reveals an HCP incidence of 11.57%, with multifactorial analysis identifying several independent risk factors for this complication, including BMI, DVT, FB, use of dehydrating agents, duration of limb immobilization, and SII. The nomogram, constructed based on these six factors, demonstrated commendable performance metrics, achieving an AUC value of 0.842 (95% CI: 0.7931–0.8907), with a sensitivity of 72.1% and specificity of 87.8%. Importantly, both internal and external validation procedures underscored the robust consistency and reliability of this predictive model.

In a retrospective study of 1125 patients with tibial diaphyseal fractures, Babak Shadgan et al. found that the incidence of ACS was 7.73%, which was considerably lower than the 11.57% incidence of HCP in pilon fracture patients in the present study [17]. On one hand, such findings could be attributed to the inclusion of more patients due to the broader diagnostic criteria of HCP compared to ACS. On the other hand, it is plausible that the soft tissue surrounding the ankle joint is inherently dense, potentially predisposing it to the development of high compartment pressures following fracture. At the same time, in the TQP database, Carl Laverdiere et al. found that 0.7% of all the foot fractures required fasciotomies [18]. However, ankle fractures were not included in the study.

In contrast to simple ankle fractures, pilon fractures typically occur due to high-energy trauma involving substantial axial force. This force often results in the bursting of the tibial plafond over the talus [19]. Many physicians contend that high-energy trauma should be considered a risk factor for ACS. This belief stems from the understanding that the extensive soft tissue damage associated with high-energy transfer is likely to lead to increased necrosis, hypoxia, lactic acidosis, capillary leakage, and interstitial fluid accumulation, culminating in compartment swelling [20]. Therefore, this study specifically focused on patients with pilon fractures resulting from high-energy trauma to the ankle. Through clinical data analysis, it confirmed the decompressive effect of FB on HCP and, for the first time, identified the SII as a potential key indicator for predicting the development of HCP.

The popular belief is that ACS is more likely to occur in young males [17]. Park et al. [21] identified age as a significant risk factor, proposing that young males, owing to their typically larger muscle bulk, may also possess thicker fascia and inter-muscular septa, attributed to a higher collagen content. As such, even a slight increase in compartment volume could lead to a rapid rise in pressure. However, the present study findings contradict this assumption, revealing that women were equally susceptible to developing HCP following pilon fractures.

Undoubtedly, injuries affecting multiple anatomical sites can significantly disrupt the body’s homeostasis, triggering what is often referred to as a “chemical storm” characterized by systemic inflammatory response syndrome (SIRS) or compensatory anti-inflammatory response syndrome (CARS). Endothelial damage has been closely associated with the occurrence of ACS in such scenarios [22]. At the same time, SII was also identified as a significant risk factor for HCP in the present study. Several scholars have found that a single cell component or blood biochemical index does not have the optimal predictive ability. Therefore, the combined ratio of various indicators, such as neutrophil to lymphocyte ratio and platelet to lymphocyte ratio, has garnered significant attention in research. These composite indicators may offer greater value than individual cellular components or biochemical markers. SII, as a novel marker, incorporates lymphocytes, platelets, and neutrophils, enabling a more balanced and comprehensive evaluation of the immune and inflammatory response within the human body. Consequently, it holds more predictive value compared to previous single or dual-cell component ratios [23]. SII has unique advantages over other biomarkers, such as neutrophil-to-lymphocyte ratio, platelet-to-lymphocyte ratio, and C-reactive protein. Studies have indicated that SII appears to be less influenced by fluid load compared to the neutrophil to lymphocyte ratio and platelet to lymphocyte ratio [24]. Monitoring changes in cytokines in FB fluid and plasma, it was observed that different cytokine activity states occurred during different periods by Hou’s team. As HCP develops, there is a shift in the systemic inflammatory state from increased to decreased [25]. Given that most patients included in the present study were in the acute phase of trauma, it was observed that higher SII can serve as one of the indicators for early prediction of HCP. Such findings align with the results of previous studies.

While there is a risk of ACS associated with any form of pilon fracture, an open fracture is commonly perceived to have effectively decompressed the compartments, thus reducing the likelihood of ACS occurrence. The auto-decompression phenomenon observed in high-grade open fractures is hypothesized to create an effect akin to that of a fasciotomy [15, 20]. Nonetheless, the present study shows that ACS is just as likely to occur in open fractures as it is in closed fractures. These results are similar to those found by Park et al. [21].

In addition to SII, BMI, DVT, duration of limb immobilization, absence of FB, and absence of dehydrating agents were also considered as potential risk factors.This selection was driven by the dense nature of the tissue around the ankle joint, with each factor possibly affecting the pressure within the local fascial compartment. Hou’s team conducted an in-depth study of HCP. Their findings suggested that fracture blisters might signify a decrease in intrafascial pressure following traumatic injury. This pressure decrease can prevent the harmful cycle of ischaemia and hypoxia triggered by further elevation of intrafascial pressure, thus averting the development of severe intrafascial hypertension [26–28]. Simultaneously, Varela et al. reported that blister formation serves as one of the mechanisms for alleviating abnormally elevated pressure within a compartment [29]. In instances of high-energy fractures, DVT plays a significant role in impaired circulation, consequently elevating pressure within the fascial compartment and heightening the likelihood of HCP. Additionally, Due to the increased subcutaneous adipose tissue, individuals with obesity are at a higher risk of developing HCP following limb injuries compared to those with normal weight; this also explains why a high BMI is considered one of the risk factors for HCP.Prolonged use of limb fixation methods, including plaster casts, bandages, and splints, can result in fascial compartment compression and a decrease in content volume, which are recognized as common risk factors for HCP [30].Conversely, the use of dehydrating agents may alleviate pressure within the fascial cavity, thereby reducing the incidence of HCP. Research indicates that the application of dehydrating agents can markedly enhance the prognosis for patients with ACS [31].

Nomograms, commonly utilized in clinical predictive model research [32–35], leverage the predictive value of individual risk factors to present final predictions visually. In the present study, a nomogram aimed at aiding clinicians in assessing the risk of HCP in recently admitted patients with pilon fractures was successfully developed and validated. The nomogram incorporates six predictors derived from routinely collected clinical data, readily available within a few hours of admission. The utilization of the nomogram expedites the identification of patients at an elevated risk of HCP. Clinicians can readily ascertain the predictive probability for each variable by drawing vertical lines corresponding to the variable outcomes and then aggregating these values to calculate the associated risk.

The present study offers notable scientific insights, including the formulation of a nomogram and its thorough validation through internal assessments. Nevertheless, it is crucial to recognize several limitations. Firstly, the present study’s retrospective nature inherently introduces the risk of selection bias, which cannot be avoided. Secondly, certain variables that could potentially influence the incidence of HCP were either not recorded or not measured, such as the duration of limb immobilization. Thirdly, there is an urgent need to clarify the diagnostic criteria for HCP. Although the STIC Monitor was used in the present study, significant discrepancies in measured results of fascial compartment pressure persisted among patients. Additionally, the clinical data used to construct and validate the nomogram was derived from a single healthcare centre. As such, caution is warranted when generalizing the findings to more diverse populations and regions. It is imperative to underscore the importance of prospective observational studies that encompass data from multiple centres. Such an approach would facilitate a more comprehensive evaluation of the clinical applicability of the proposed model.

## Conclusion

In conclusion, the present study reveals that, compared with tibial diaphyseal fractures, pilon fractures are more prone to HCP.At the same time, several key factors emerged as significant predictors for postoperative HCP in individuals afflicted with isolated pilon fractures. These factors encompass BMI, DVT, occurrence of FB, use of dehydrating agent, duration of limb immobilization, and SII.In order to enhance the precision of risk assessment, a nomogram grounded in these identified predictors was meticulously devised.The incorporation of the proposed nomogram into clinical practice provides healthcare professionals with a valuable instrument for promptly assessing the risk of HCP in hospitalized patients, particularly those diagnosed with pilon fractures. In additon, this study found for the first time that SII is an independent risk factor for HCP, which provides a basis for clinical research on fascial immunology in the future.

## Data Availability

Yes.

## Abbreviations

ACS: Acute Compartment Syndrome
AUC: Area Under the Curve
ACL: Anterior Cruciate Ligament
ALB: Albumin
ALP: Alkaline Phosphatase
AST: Aspartate Aminotransferase
ALT: Alanine Transaminase
BMI: Body Mass Index
BAS: Basophil
C-index: Conformance Index
Ca: Calcium
CHE: Cholinesterase
CK: Creatine Kinase
CREA: Creatinine
DVT: Deep-Vein Thrombosis
DCA: Decision Curve Analysis
DBIL: Direct Bilirubin
EOS: Eosinophil
FB: Fracture Blister
GLOB: Globulin
GLU: Glucose
HCP: High Fascial Compartment Pressure
HCT: Hematocrit
HGB: Hemoglobin
LYM: Lymphocyte
LDH: Lactic Dehydrogenase
MON: Monocyte
MPV: Mean Platelet Volume
NETs: Neutrophil Extracellular Traps
NEU: Neutrophil
PLT: Platelet
ROC: Receiver Operating Characteristic Curve
RBC: Red Blood Cell
SII: Systemic Immune-inflammation Index
TG: Triglyceride
TC: Total Cholesterol
TCO2: Total Carbon Dioxide
TP: Total Protein
UREA: Ureophil
UA: Uric Acid
WBC: White Blood Cell
5Ps: Pain, Paresthesia, Paralysis, Pulselessness and Pallor
